# Supplementary material for: Gymnema Sylvestre Supplementation Restores Normoglycemia, Corrects Dyslipidemia, and Transcriptionally Modulates Pancreatic and Hepatic Gene Expression in Alloxan-Induced Hyperglycemic Rats
Source: Metabolites. 2023 Apr 4;13(4):516. doi: 10.3390/metabo13040516 (PMC10142569; doi:10.3390/metabo13040516)
Supplement: Supplementary file 1 [file metabolites-13-00516-s001.zip › metabolites-2122448-supplementary.pdf]

Table S1. Primer sequence for RT-qPCR

**Gene**	**Primer sequence**	**Annealing temp**	**Amplification Cycles**
Actb-F	TCCTGAGCGCAAGTACTCTGT	**55°C**	**30**
Actb-R	CTGATCCACATCTGCTGGAAG	**55°C**	**30**
Ins1-F	GCAAGCAGGTCATTGTTTCAAC	**56°C**	**28**
Ins1-R	AAGCCTGGGTGGGTTTGG	**56°C**	**28**
Ins2-F	GTGGTTCTCACTTGGTGGAAGCTC	**55°C**	**30**
Ins2-R	CTCCAGTGCCAAGGTCTGAAGGT	**55°C**	**30**
GCK-F	GGCCACCAAGAAGGAAAAGGT	**55°C**	**30**
GCK-R	CCTCTCCCACTTTGACCAGCA	**55°C**	**30**
Irs1-F	AGAGTGGTGGAGTTGAGTTG	**57°C**	**30**
Irs1-R	GGTGTAACAGAAGCAGAAGC	**57°C**	**30**
Irs2-F	GGATAATGGTGACTATACCGAGA	**55°C**	**36**
Irs2 -R	CTCACATCGATGGCGATAAGTT	**55°C**	**36**
GLUT2-F	TACTGGCACATCCTACTTGG	**55°C**	**35**
GLUT2-R	ATTCCAGAGAACTGCTGAGC	**55°C**	**35**
PDX-1-F	CGTAGTAGCGGGACAACGAG	**56°C**	**38**
PDX-1-R	CCCGAGGTTACGGCACAAT	**56°C**	**38**
MafA-F	CGCACCCGACTTCTTTCTGT	**55°C**	**36**
MafA-R	CTCAGAGTCCGAACCGAGG	**55°C**	**36**
Nfkb1-F	TAGCCACAGAGAGATGGAGGAG	**60°C**	**36**
Nfkb1-R	CCGAGTCGCTATCAGAGGTA	**60°C**	**36**
Nrf2-F	CACATCCAGACAGACACCAGT	**58°C**	**37**
Nrf2-R	CTACAAATGGGAATGTCTCTGC	**58°C**	**37**
CAT-F	GCGAATGGAGAGGCAGTGTAC	**52°C**	**30**
CAT-R	GAGTGAGTTHTCTTCATTAGCACTG	**52°C**	**30**
SOD1-F	GCAGAAGGCAAGCGGTGAAC	**52°C**	**30**
SOD1-R	TAGCAGGACAGCAGATGAGT	**52°C**	**30**
SOD2-F	CTGAGGAGAGCAGCGGTCGT	**58°C**	**30**
SOD2-R	CTTGGCCAGCGCCTCGTGGT	**58°C**	**30**
Pax6-F	AAGAGTGGCGACTCCAGAAGTTG	**56°C**	**36**
Pax6-R	ACCACACCTGTATCCTTGCTTCAGG	**56°C**	**36**
FoxO1-F	GTGCCCCAGGACTCTTGAAA	**58°C**	**34**
FoxO1-R	ACTGTTGGGTTGAGCCACTC	**58°C**	**34**
FoxA2-F	AAGGGAAATGACAGGCTGAGTGGA	**60°C**	**32**
FoxA2-R	TGTGGAACTCTGGCATTCTAGCCA	**60°C**	**32**
Foxk1-F	CTACCCCAACAATGCACCTT	**60°C**	**32**
Foxk1-R	GAGGACTTGCTGGACAGGAG	**60°C**	**32**
Chrebp a-F	CGACACTCACCCACCTCTTC	**60°C**	**30**
Chrebp a-R	TTGTTCAGCCGGATCTTGTC	**60°C**	**30**
Srebp1c	GGAGCCATGGATTGCACATT	**60°C**	**32**
Srebp1c	AGGCCAGGGAAGTCACTGTCT	**60°C**	**32**
